# Sudden Death by Shock after Surgical Operations

**Published:** 1872-12

**Authors:** 


Sudden Death by Shock After Surgical Operations.—Prof. Fischer, of Breslau, (*Berliner Klinische Wochenshcrift, No.* 24, 1871) gives the history of a case of sudden death which occurred at his clinic immediately after a resection of the superior maxilla of a rather debilitated man of 56 years old. Chloroform had not been used in the operation, and an autopsy revealed no perceptible changes anywhere ; hence nervous shock remained as the only cause to account for the sudden death.

Dr. Bertrand, of Schlangenbad, adds in No. 35 of the same Journal of 1872, some more cases of the same kind to those already on record, principally for the purpose of cautioning the profession against the error of ascribing sudden death always to the use of chloroform in operations.

His first case is one which was related to him by the celebrated Prof. Simpson, of Edinburgh. It occurred soon after the discovery of chloroform, when Simpson already was its warm and very nearly its only advocate at that city. He had persuaded Prof. Miller to use it in an operation for strangulated hernia ; the plan, however, was abandoned, and no chloroform was used, very much to the future satisfaction of Prof. Simpson, as the patient expired suddenly under the hand of Prof. Miller, after he had made the cutaneous incision while he was engaged in dividing the layers of connective tissue surrounding the hernial sac. The post-mortem examination furnished no information as to the cause of his death.

If chloroform had been used in this case, as it was intended—no matter how small the quantity—it would have been considered the cause of death, and as Simpson remarked, its fate as an anæsthetic would have been sealed forever.

In another case in his own practice, where so small a quantity of chloroform had been used, that the patient became fully conscious at the first incision, which was made to execute herniotomy for the relief of a strangulated hernia ; a sudden collapse took place, and notwithstanding the usual method was applied to maintain respiration and circulation, the subject of the operation, an old man, died within a few hours afterwards.—*Berliner Klinische Wochenschrift, No.* 35, 1872. R.

The Breath of Diabetic Patients is said to have a specific odor similar to that of some habitual drunkards, that is acid and slightly alcoholic. M. Debouée first reported the fact before the Société de Chirurgie, and M. Guéneau de Mussy confirmed it in the *Gazette Hebdomadaire* The latter based his diagnosis several times on the presence of this odor, and observed that it decreased with the decrease of sugar in the urine. —*Berliner Klin. Wochenschrift, No.* 35, 1872.

